# Nutrition in HNSCC: is it a matter for oncologists? The role of multidisciplinary team—a narrative literature review

**DOI:** 10.3389/fonc.2024.1430845

**Published:** 2024-07-03

**Authors:** Nerina Denaro, Claudia Bareggi, Barbara Galassi, Giada Beltramini, Demi Wekking, Michele Proh, Tullio Ibba, Cinzia Solinas, Ornella Garrone

**Affiliations:** ^1^ Oncologia Medica Fondazione Istituto di Ricovero e Cura a Carattere Scientifico (IRCCS) Ca’ Granda, Ospedale Maggiore Policlinico Milano, Milan, Italy; ^2^ Chirurgia Maxillofacciale Fondazione IRCCS Ca’ Granda, Ospedale Maggiore Policlinico Milano, Milan, Italy; ^3^ Academic Medical Centre, Amsterdam UMC, University of Amsterdam, Amsterdam, Netherlands; ^4^ Otorinolaringoiatra Fondazione IRCCS Ca’ Granda, Ospedale Maggiore Policlinico, Milan, Italy; ^5^ Medical Oncology Department, Azienda Ospedaliera Universitaria (AOU) Cagliari, Policlinico Di Monserrato (CA), Monserrato, Italy

**Keywords:** malnutrition, sarcopenia, cachexia, HNSCC, multidisciplinary team

## Abstract

**Background:**

Malnutrition, defined as weight loss and unsatisfactory nutrient intake, is very common in patients with head and neck squamous cell carcinoma (HNSCC) in either the early or palliative setting. Despite increased awareness, nutritional programs are not yet adequately implemented in these patients. There are several reasons for this delay: differences in composition, the expertise of the multidisciplinary teams involved in HNSCC patients’ treatment, and economic and network resources conditioning faster or slower nutritional supply delivery. This situation affects the outcomes and the quality of life of HNSCC patients.

**Materials and methods:**

We investigated available literature about nutritional support in HNSCC patients and its impact on outcomes, prognosis, and quality of life, and we focused on the role of the multidisciplinary team. We considered 8,491 articles, and after excluding duplicates and manuscripts not written in English, 1,055 were analyzed and 73 were deemed eligible for the present work.

**Results:**

After the literature review, we can state that malnutrition, sarcopenia, and cachexia are associated with systemic inflammation and closely correlated with poor outcomes. An evaluation of the nutritional status of the multidisciplinary team before, during, and after therapy could improve patient outcomes, as the goal of the therapeutic approach is widely designed.

**Conclusions:**

We suggest that the treatment workflow definition is fundamental and propose a tailored nutritional approach that could benefit HNSCC patients’ outcomes and quality of life. These results could be achieved by a multidisciplinary team.

## 1 Introduction

Head and neck squamous cell carcinoma (HNSCC) is the sixth most common neoplasm worldwide and is represented by a complex group of rare tumors arising from the upper aerodigestive mucosa tract (1). The standard of care in the early stage is surgery (S) or radiotherapy (RT), while in the locally advanced stage, a multidisciplinary approach is required with either S followed by concurrent chemoradiotherapy (CRT) or chemotherapy (CT) alone (2).

The latter is the standard of treatment for locally advanced (LA) HNSCC not accessible to surgical resection.

Most HN cancer patients are malnourished due to difficulties correlated to the primary tumor site and RT or S effects on anatomy and functions (3).

Common problems include difficulty in chewing and swallowing, loss of appetite, nausea, trismus and edentulism.

The location of the tumor, a previous history of alcoholism, an impaired immune response, and acute and late treatment toxicities contribute to malnutrition.

Heterogeneity in malnutrition definition, high levels of variability among HNSCC patients, and treatment modalities contribute to difficult diffusion and standardization of adequate nutrition management.

According to the increasing relevance of nutritional status in the management and treatment outcomes of head and neck cancer patients, we reviewed available literature according to the following topic:

1. Malnutrition, sarcopenia, and cachexia definitions in HNSCC;
2. The role of the multidisciplinary team in promoting nutrition;
3. Challenges and unmet needs in locally advanced and recurrent/metastatic settings; and
4. The role of the microbiome.

The goal of this narrative review is to underline the role of different specialists in supporting dieticians and nutritionists to manage patients with either early-stage LA HNSCC or recurrent metastatic (RM) HNSCC.

Therefore, we will not consider recommendations for nutritional and metabolic management such as the ketogenic diet or fasting and calorie restriction in patients with HNSCC.

Moreover, we analyzed the nutrition in the HNSCC patients’ journey: in the locally advanced setting, the curative role of CRT and its acute and late side effects may conditionate the nutritional status of patients for the rest of their lives. In the recurrent metastatic setting, swallowing impairment and sarcopenia status may condition the outcome.

## 2 Materials and methods

A search of the literature was conducted through the PubMed/MEDLINE database with the following search terms: “Head and Neck Neoplasms”(Mesh) AND [“Nutritional Status”(Mesh) OR “Nutrition Therapy”(Mesh) OR “Nutrition Assessment”(Mesh) OR “Immunonutrition Diet”(Mesh) OR “Sarcopenia”(Mesh) OR “Cachexia”(Mesh) OR “Deglutition Disorders”(Mesh)]. Rayyan website free tool was employed to select eligible papers.

The search was limited to English-language articles published from 2009 to September 2023. A total of 8,491 papers were considered.

Duplicate papers, articles not in English, papers on esophageal cancer, and case reports were excluded. In the end, 73 were included in the review.

Search was performed by two authors [N.D. and C.B], who independently selected eligible papers. In the Supplementary Material, a flow diagram of selection is reported.

## 3 Results

### 3.1 Malnutrition, sarcopenia, and cachexia in HNSCC: is there an appropriate definition?

Malnutrition, sarcopenia, and cachexia are closely related and commonly under-recognized and undertreated. Sarcopenia impacts severe HNSCC patients older than 65 years, as elderly patients develop “physiologically” lean mass wasting.

Despite increasing awareness of the importance of nutrition in oncological patients and clinical practice highlights and guidelines of the European Society for Clinical Nutrition and Metabolism (ESPEN) and American Society for Parenteral and Enteral Nutrition (ASPEN), surveys demonstrated still a disagreement not only on the management but also on the definition of malnutrition.

There are several obstacles to adherence to nutritional guidelines in clinical practice. Neither energy nor protein intake prescriptions are followed. The most important barriers included institutional factors, individual provider behavior, and delays in the start and prescription of enteral nutrition. Barriers can vary widely from one hospital to another and according to local and socioeconomic contexts (4). Approximately one-third of physicians recognize key criteria of cancer cachexia and malnutrition; of them, approximately 15% do not utilize a standardized definition, and 50% of oncologists do not weigh patients at each visit (5–9).

Malnutrition may be defined as low body mass index (BMI) <20 kg/m^2^, involuntary weight loss (>5%–10% of body weight) over the past 6 months, and decreased nutritional intake. However, a deficit of energy, a deficit of protein, and decreased fat-free mass are also considered key elements (10).

Wj Evans et al. in 2008 defined cachexia as weight loss greater than 5% over the prior 12 months in the presence of cancer and three associated conditions: decreased muscle strength, fatigue, anorexia, low fat-free mass, or abnormal biomarkers (albumin, C-reactive protein, total protein, white blood cell, hemoglobin, and transferrin) (11).

Nutritional and hematologic markers correlate with a worse prognosis.

There is a continuum among malnutrition pre-cachexia, cachexia, and refractory cachexia (12).

Noteworthy, inflammatory markers (neutrophil/lymphocyte ratio) may indicate the severity of malnutrition and correlate with prognosis.

Definitions of sarcopenia also differ among different studies, as follows: European Working Group on Sarcopenia in Older People (EWGSOP2) [grip strength <27 kg and appendicular lean mass (ALM) index <7.0 kg/m^2^], Sarcopenia Definitions and Outcomes Consortium (SDOC) (grip strength <35.5 kg and gait speed <0.8 m/s), and Modified SDOC (grip strength <35.5 kg and gait speed <1.0 m/s) (13).

The prevalence of malnutrition and sarcopenia in patients with cancer, which depends on the tumor stage and site, may be up to 80%. Even when a state of malnutrition is detected, corrective measures are often not adequately implemented (5).

Several tools have been developed to assess malnutrition such as the Malnutrition Universal Screening Tool (MUST), the Nutritional Risk Screening 2002 (NRS 2002), the Short Nutritional Assessment Questionnaire (SNAQ), and the Functional Oral Intake Scale (FOIS).

MUST is a simple, rapid, and easy method to screen patients and has been proven to be reliable and valid (14).

It aims to identify those at risk by incorporating the following:

• Current weight and body mass index calculated by height and weight,
• History of recent unintentional weight loss, and
• Likelihood of future weight loss.

Nutrition Risk Screening 2002 (NRS-2002) is a simple and well-validated tool that incorporates pre-screening with four questions. Developed approximately two decades ago, it considers a screening with surrogate measures of nutritional status, and it integrates static and dynamic parameters and data on the severity of the disease (stress metabolism). If there is the presence of one positive answer (BMI < 20.5, weight loss in the past 3 months, food intake reduction in the last week, and acute illness), the NRS-2002 is positive and screening is required (15).

For each parameter, a score from 0 to 3 can result. Age over 70 years is considered a risk factor and is included in the screening tool as well, giving 1 point. A total score of ≥3 points means that the patient is at risk of malnutrition or is already malnourished, and therefore, nutritional therapy is indicated. The NRS-2002 has been assessed and validated in hundreds of studies, including randomized controlled trials, and has been shown to be very reliable if administered by trained staff (15).

Simplified Nutritional Appetite Questionnaire (SNAQ) is a tool based on 26 questions related to eating and drinking difficulties, defecation, condition, and pain; it is widely reproducible (16).

The FOIS is a validated, 7-point, ordinal scale that indices the type of oral intake (food and liquid) that an individual is able to consume and his or her reliance on a feeding tube for nutritional intake (17).

Other institutions use the Patient-Generated Subjective Global Assessment (PG-SGA), which evaluates the history of weight loss, presence of symptoms, and nutritional intake and divides patients into three categories (well-nourished, suspected of being malnourished, and severely malnourished) (18).

Swallowing disorders must be investigated as nutritional status: the M.D. Anderson Dysphagia Inventory (MDADI) and Eating Assessment Tool (EAT)-10 questionnaire are commonly used.

The first is a questionnaire currently used for the assessment of dysphagia-related disability in patients with HN cancer. Its consistency and test–retest reliability are high in this population (19).

In our review, we did not focus on the validity of a screening tool over another one.

The experience of the multidisciplinary team (MDT) is needed to define the prognosis and the life expectancy, avoiding the exclusion of curative treatment patients with a low BMI and severe malnutrition and identifying the goals of nutritional management.

Each team could use, on the basis of its own expertise, a different test to assess the urgency and the modality to support the patients (20).

**Figure 1:**
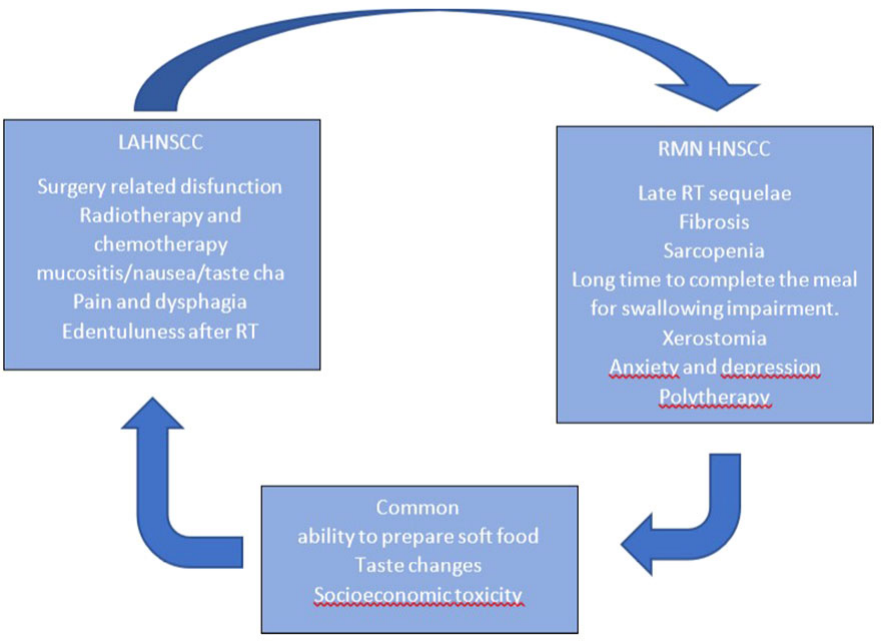
Nutritional characteristics in LA HNSCC /RM HNSCC setting.

In **Figure 1**, we present the workflow of the multidisciplinary team.

### 3.2 The role of the MDT in promoting nutrition

In head and neck cancer, the role of MDT is fundamental not only when surgical versus no surgical approach has to be decided. HN cancer unit is important in the management of nutrition in these patients. MDT members consider treatment-related side effects and their impact on the nutritional program compliance. Nutritionists and dieticians are informed in the multidisciplinary meeting about the prognosis as well as the CT- and RT-related symptoms to personalize their approach. For example, a patient with severe dysphagia receiving analgesic transmucosal opioids may have nausea and trouble integrating oral nutritional supplements (ONSs). In Italy, a multidisciplinary approach is warranted for most cancer treatment patients; however, the composition of MDT and the flow from diagnosis to therapies vary enormously among different regions. For example, the absence of networking conditions late care of outpatients with swallowing impairment or malnutrition.

In the absence of an MDT discussion, the risk of missing relevant factors is high. In addition to that, the MDT should be involved in the patients’ journey from the initial diagnosis to palliative care. Furthermore, every dysfunction in swallowing and malnutrition sign or symptom should be assessed in a pre-treatment evaluation and during and after treatment with speech-language pathologists and nutritionists (21, 22).

While it is easier to organize nutritional supplies such as immune–nutrition before surgery, as it is widely accepted and improves the duration of recovery, its role in the chronic supportive care in LA/RM HNSCC is known but not widely implemented (5).

Moreover, swallowing impairment, surgery sequelae, and acute and late toxicities of radiotherapy with or without concurrent chemotherapy may impact HNSCC patients’ nutrition.

Among randomized controlled trials (RCTs), acute effects such as mucositis, pain, dysphagia, and taste challenges are very common. Conversely, late effects include trismus (sometimes it is also challenging to introduce a little spoon into the mouth), xerostomia, and fibrosis.

Radiation-induced nausea and vomiting (RINV) is a frequent albeit neglected side effect of RT that can lead to delays in treatment with serious consequences on cure rates. Modern techniques have increased this risk, which correlates with radiation dose on the dorsal vagal complex (23).

Cisplatin-based CT may cause mild nausea, vomiting, loss of the ability to taste food, hiccups, dry mouth, dry skin, and dehydration (24).

Swallowing dysfunction is common in patients who present with HNSCC, and failure to recognize this condition can lead to worsening malnutrition or aspiration pneumonia (25–28).

Muscle-targeted oral nutritional supplementation (MT-ONS), namely, a whey-protein-based, leucine- and vitamin D-enriched formula, was found to impact sarcopenia, with efficacy in increasing the muscle mass and strength, as well as the physical performance, versus isocaloric placebo or standard practice. A higher benefit was obtained for those who added physical exercise and protein supplementation (29, 30).

In the last 5 years, the Italian Intersociety Working Group (WG) for Nutritional Support in Cancer Patients confirmed a major awareness in the oncology teams but still few concrete strategies aimed at facing the nutritional care gap in this population. Recently, the group provided an update on the 2016 WG practical recommendations, highlighting the need for active improvement and implementation (31).

HNSCC patients referred to palliative medicine are burdened by multiple physical psychological substance use and social challenges.

Nevertheless, nutritional goals should be shared with the patients and caregivers also in palliative and end-of-life care (32).

Moreover, HN cancer physicians should be aware of anxiety, neuroticism, and stress in these patients. It has been widely demonstrated that satisfactory communication (sharing treatment and nutritional goals with benefits and weaknesses) minimizes anxiety and distress in patients (33–36).

Among the advantages of screening and on-time support (versus delayed approach) are better progression-free survival (PFS), overall survival (OS), and quality of life (QoL); higher nutrition intake and better nutritional status and impact on quality of life; fatigue reduction; rapid return to work; higher performance status; treatment completion and few treatment interruptions; and reduction in treatment toxicities and unplanned hospital admission.

The benefits of a prophylactic endoscope tube (PEG) include improvement in QoL, maintenance of weight and lean mass, and reduction in hospitalization. On the contrary, longer duration of reliance on PEG, higher frequency of esophageal stricture, laryngeal irritation, gastroesophageal reflux, and rare risk of abdominal seeding were described (5).

### 3.3 Challenges and unmet needs in the locally advanced setting and recurrent metastatic

Recently, there has been major awareness of the predictive and prognostic role of nutritional status for LA HNSCC, as reported above; however, few data are available in RM HNSCC.

**Figure 2:**
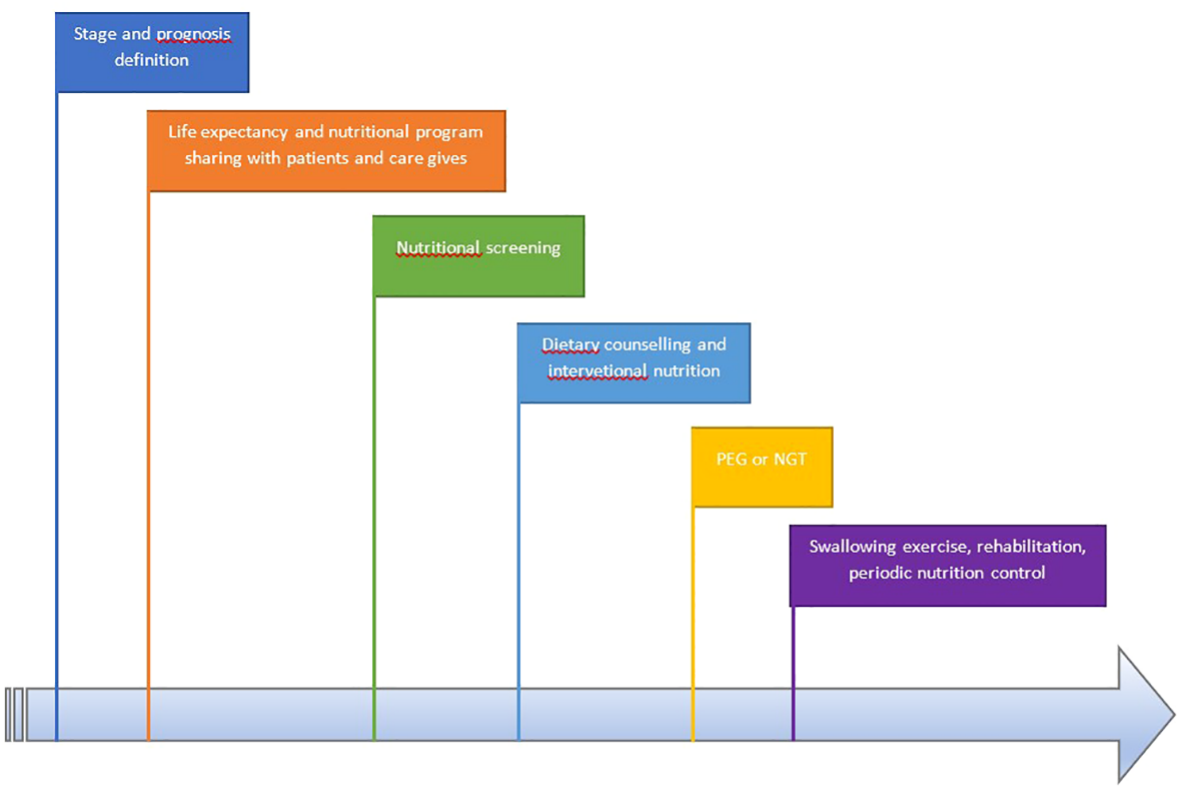
Workflow of the multidisciplinary team.

In **Figure 2**, we report nutrition-related characteristics in different settings. A different approach is required for patients with short versus long life expectancy: for those with short life expectancy, dietary counseling, treatment, and disease-related adverse event counseling (e.g., nausea, diarrhea, vomiting, and constipation) are recommended.

If an unfavorable prognosis is expected, strict control of blood sugar and cholesterol levels may no longer be as important as maintaining body weight in patients with heart disease or diabetes comorbidities.

If a good prognosis is probable, it is recommended to encourage physical activity, short-chain fatty acid introduction, and probiotic and prebiotic balance (5).

First, data on HNSCC nutritional supplementation were controversial.

D. Jin reported that parenteral nutritional support was associated with significant stimulation of tumor proliferation as measured by an increase in the percent of tumor cells in the S phase, DNA content, and DNA index (37).

A secondary analysis of Radiation Therapy Oncology Group (RTOG) 90–03, a prospective randomized trial evaluating four definitive RT fractionation schedules in patients with LA HNSCC, evaluated data on nutritional support before [basic nutritional support (BNS)] during or after RT. Patients receiving BNS experienced significantly less weight loss by the end of treatment and less grade 3 to 4 mucositis than patients not receiving BNS, but BNS was a highly significant independent prognostic factor for increased locoregional failure and death. NS is associated with not only improved patient outcomes but also inferior cancer outcomes (38).

Other authors demonstrated that protein supplementation failed to reduce sarcopenia. Protein supplementation works only if it is associated with exercise (39).

Currently, the nutritionist’s role includes counseling and nutritional supplement prescription (oral/enteral or intravenous), energy/protein support, avoiding potentially harmful refeeding syndrome, encouraging anti-catabolic and anti-inflammatory ingredients in diet (e.g., essential amino acids or high dose leucine, fish oil, and source of long-chain omega-3 fatty acids, omega-3 fatty acid, arginine, and nucleotides).

The role of prophylactic gastrostomy is uncertain. A Cochrane review demonstrated no benefit over nasogastric tube (40). Advantages of tube feeding include improved quality of life and lower frequency of severe weight loss and hospital admissions. On the contrary, a longer duration of reliance on the feeding tube, a higher incidence of esophageal stricture, and laryngeal dysfunction have been reported as well as a higher cost. Parenteral nutrition improves QoL and nutrition parameters if used for a short period (5).

A.C.H. Willemsen developed a tool to predict the tube feeding dependency considering pre-treatment weight change, texture-modified diet at baseline, Eastern Cooperative Oncology Group (ECOG) performance status, tumor site, N classification, and RT dose to the parotid gland and oral cavity (41).

Currently, nutritional status is correlated with prognosis, and it is predictive of immunotherapy response. Exercise and nutritional programs should be shared in MDT; moreover, tooth extraction could not be recommended in all patients with oropharyngeal cancer, as it reduces abscesses but increases body weight loss and tube feeding dependence (42).

Poor OS was also associated with a greater decrease in the pre-treatment BMI trend. An association was found between BMI (normal) and immune checkpoint inhibitor (ICI) treatment response and OS in melanoma and non-small cell lung cancer (NSCLC) patients (43).

In a population of 352 patients with non-metastatic laryngeal (146) and oropharyngeal (206) cancer treated with definitive RT, sarcopenia based on muscle areas at the L3 level on CT scan correlated with the outcome. Patients with higher compliance to the nutritional program have a reduction in death risk of approximately 27% and a reduction in disease progression risk of approximately 31%. Higher pre-treatment BMI was also associated with a lower risk of death and disease progression (44).

The negative effect of sarcopenia in HNSCC is well documented (45, 46). Approximately one-third of HNSCC patients may present with sarcopenia. Pre-treatment sarcopenia is associated with significantly worse outcomes (47–49).

Patients with sarcopenia are more than twice as likely to suffer short-term treatment-related toxicity when undergoing curative-intent head and neck cancer (HNC) treatment (50).

Sarcopenic patients had a worse treatment outcome, namely, poorer disease-free survival, more toxicities, and more treatment gaps. An Indian tertiary care hospital reported the prognostic impact of sarcopenia by calculating skeletal muscle index (SMI) using CT images of the C3 vertebrae on 300 patients, and the authors formulated a cutoff value of 32 cm^2^/m^2^, which correlates with the outcome. As per the receiver operating characteristic (ROC) curve, patients with SMI > 32 cm^2^/m^2^ fared better than those with SMI < 32 cm^2^/m^2^ (46).

Pre-CT nutritional status and neutrophil/lymphocyte ratio (NLR) influence the functional QoL, strength, and response. Guller et al. demonstrated that the prognostic nutritional index scores but not body mass index correlated with overall survival progression-free survival and immunotherapy response. The prognostic nutritional index (PNI) is calculated using the albumin level reflecting nutritional status and lymphocyte count reflecting immune status. It is considered a prognostic biomarker for its correlation with cancer-specific survival. Moreover, elevated PNI was associated with a significantly lower risk of death in patients candidate for PD-L1/PD-1 inhibitor monotherapy (51).

The use of ONS reduced the need for changes in scheduled anti-cancer treatments [i.e., for RT and/or systemic treatment dose reduction or complete suspension, hazard ratio (HR) = 0.40 (95% CI, 0.18–0.91), p = 0.029] (52).

Among the biomarkers, neutrophil/lymphocyte ratio is widely available, and NLR ≥ 4.5 at pre-treatment status significantly correlated with short OS and PFS and malnutrition status. High NLR in peripheral blood was significantly correlated with low lymphoid cell and high tumor-associated macrophage counts in tissues, especially myeloid-to-lymphoid cell ratios, suggesting an association between circulating and intratumoral immune complexity profiles (53).

A correlation among albumin levels, Hb levels, and BMI has been demonstrated in several reports (54–56).

The prevalence and consequences of nutrition impact symptoms are substantial among head and neck cancer survivors beyond the acute phase of cancer treatment. Oncology clinicians should continuously monitor and manage these symptoms throughout the cancer continuum (57).

In long-term survivors undergoing swallowing rehabilitation, the time to have a meal is long and affects psychosocial function and quality of life. A Mediterranean diet is actually the most approved approach in patients without impairment of swallowing (58).

Consumption of a diet rich in vegetables, fruits, fish, legumes, and whole grains reduces proinflammatory cytokines. Adherence to a Mediterranean diet reduces cancer-specific mortality (59).

Moreover, we need to remember that feeding an incurable ill patient does not mean only feeding him/her but also involves a series of important emotional aspects. In **Figure 2**, we summarize the characteristics of early- and late-stage diseases regarding nutrition. In our experience (based on patients’ reported outcomes), the psychological aspect of food presentation, texture, and palatability is reported to be of great importance by the patients.

### 3.4 The implication of the immune system and microbiome

Inflammation correlates with metabolic alteration, and cytokine-mediated (IL-6, IL-2, IL-18, and aTNF) responses induce loss of lean body mass and diminished function and acute-phase protein (APP). Metabolic changes include muscle mass wasting, liver metabolism changes, fat use and depletion, anorexia, and fluid shifts to the extracellular compartment. When chronic prolonged severe nutrition impairment occurs, it leads to cancer cachexia (60).

Cancer cachexia is a complex systemic catabolism syndrome characterized by muscle wasting.

Signal communications among tumor and muscle, fat, liver, heart, pancreas, and the intestinal tract aggravate the process of cachexia.

IL‐6 secreted by hepatocytes can promote muscle wasting, and the canonical IL‐6/JAK/STAT and FGF/p38 MAPK signaling pathways can cause muscle wasting and lipolysis (61).

A pre-treatment evaluation demonstrated that dietary intake correlates with cytokine levels. There were seven cytokines with significant associations involving the Alternative Healthy Eating Index (AHEI) 2010, seven cytokines with associations involving the Disability of the Arm, Shoulder and Hand (DASH) - index, and significant associations involving the low carbohydrate and other low carbohydrate indices (61). A higher score on the AHEI-2010 was significantly associated with higher odds of lower IFN-γ, IL-6, IL-10, IL-17, IL-8, TNF-α, and GRO-β. Pro-tumorigenic bacteria increase oxidative stress and DNA damage, modify neutrophil/lymphocyte ratio, reduce NK cytotoxicity, activate lymphocyte T helper 17, reduce T-cell density, and activate WNT β-catenin signaling and STAT3 signaling [Wekking D in press].

Oral mucositis is a common side effect of radiotherapy and chemotherapy. Approximately 60% of patients who undergo chemoradiotherapy develop mucositis. Oral cavity and bowel microbiota correlated with the incidence of mucositis.

During severe oral mucositis, a higher frequency of *Actinobacillus*, *Mannheimia*, and *Streptobacillus* was observed; on the contrary, for patients who developed mild mucositis, high levels of *Enhydrobacter*, *Schwartzia*, *Pseudoramibacter*, *Treponema*, *Prevotella*, *Fusobacterium*, *Porphyromonas*, *Megasphaera*, and *Cardiobacterium* were observed.

Anti-tumorigenic and pro-tumorigenic bacteria have been classified, although their role is pleiotropic, and some species are found in both the early and late phases of the disease [Wekking D in press].

Antibiotics’ effect on microbiota decreases OS in both LA and R/M HNSCC (62). We need to consider gut microbiota metabolism in patients with long life expectancy. It is recommended to suggest dietary modifications such as short-chain fatty acid (SCFA) introduction. SCFAs increase tumor-killing CD4+ and CD8+ T cells and reduce T-regs (63).

Butyrate and propionate can increase the intratumoral T cells and TNF-α and reduce histone deacetylases (HDACs), increasing chemosensitivity, tumor apoptosis, and cell growth inhibition, migration, and invasion (63).

Responders to immunotherapy also had a higher abundance of butyrate-producing microbes and higher levels of fecal and plasma SCFAs (64–66).

SCFAs (butyrate and valerate), fiber, or SCFA-producing bacteria supplements increase the intratumoral T cells, INF-γ, and TNF-α and result in the inhibition of tumor growth and improvement of anti-tumor immune response (67, 68).

For radiotherapy and chemotherapy, a higher abundance of butyrate-producing bacteria and higher levels of fecal SCFAs correlate to a better response.

Butyrate improved cell sensitivity to 5-FU by augmenting 5-FU-induced inhibition of DNA synthesis (69).

Gut microbiota shifts metabolic alteration and disease modulation. High fiber improves outcomes.

A cohort study demonstrated relative risk (RR) of 0.68 (95% confidence interval, 0.60–0.76), odds ratio (OR) of 0.57 (0.48–0.67), and HR of 0.58 (0.51–0.67) for those patients who received at least once antibiotics during 3 months before or after immunotherapy (70).

In patients candidate for curative chemoradiation, microbiota influences acute toxicity development and compliance to radiotherapy (pre-treatment low abundance of *Streptococcus*, *Staphylococcus*, and *Lactobacillus* and high abundance of *Fusobacterium*, *Haemophilus*, *Tannerella*, *Porphyromonas*, and *Eikenella* were associated with grade 2 mucositis) (71).

Antibiotic administration on 154 patients during CRT diminished PFS (HR = 1.397, p < 0.05, log-rank test) and OS (HR = 1.407, p < 0.05) (72).

Moreover, antibiotic consumption is common, as the risk of infection is quite common in HNSCC both for swallowing impairment (pneumonitis risk) and for the site of primary tumor. If an antibiotic is necessary, pieces of evidence suggest avoiding antibiotics that more perturbate the commensal microbiota such as azithromycin. The microbiome can be modified by symbiotic treatment using prebiotics and probiotics. The impact of concurrent probiotics on those who cannot avoid antibiotics is not known.

It must be stressed that both gut and oral cavity microbiota may vary according to microenvironment conditions (saliva flow, temperature, and pH) and growth conditions (saliva versus mucosa versus tonsil crypts) as well as the immune status of each patient. A shift in microbiota community pre- and post-radiotherapy is also reported (73).

## 4 Conclusions

According to available literature, multidisciplinary team involvement is important to adequately manage nutrition in both curative and palliative HNC therapy. Knowing the prognosis, timing, and severity of expected treatments’ adverse events helps dieticians to adapt the nutritional program.

Malnutrition is associated with inflammatory responses and unfavorable outcomes.

The goals of the multidisciplinary team in HNSCC management are to improve outcomes and QoL, avoid interruption in anti-tumoral treatments, increase compliance of patients with the oncologic therapies, minimize food-related discomfort, and maximize food enjoyment.

Sharing nutritional and therapeutic decisions with patients and caregivers is important to fulfill the three objectives of local control, survival, and quality of life. The heterogeneity of studies focusing on malnutrition, cachexia sarcopenia, and HNSCC patients negatively impacts diffusion and adherence to current guidelines.

There is a higher awareness of the malnutrition problem, but much work is still required. Early setting and recurrent metastatic disease require simultaneous care. Communication of the nutrition care plan among the team members and to the patient and caregivers is important; the plan of care must be involved and educated. Providing appropriate intervention strategies may decrease treatment complications and length of hospitalization.

Nutritional intervention is, therefore, a cure protocol part; we should manage intake, recommend physical exercise and psychological interventions, and support swallowing rehabilitation.

## Author contributions

ND: Conceptualization, Data curation, Resources, Writing – original draft, Writing – review & editing. CB: Methodology, Supervision, Writing – original draft. BG: Data curation, Methodology, Writing – original draft. GB: Conceptualization, Supervision, Validation, Writing – original draft. DW: Data curation, Writing – review & editing. MP: Methodology, Resources, Writing – original draft. TI: Conceptualization, Supervision, Writing – review & editing. CS: Supervision, Writing – review & editing. OG: Supervision, Visualization, Writing – review & editing.
