# Supplementary material for: Nutrition in HNSCC: is it a matter for oncologists? The role of multidisciplinary team—a narrative literature review
Source: Front Oncol. 2024 Jul 3;14:1430845. doi: 10.3389/fonc.2024.1430845 (PMC11251948; doi:10.3389/fonc.2024.1430845)
Supplement: Supplementary file 1 [file DataSheet_1.docx]

**8491** references

**- 2616** excluded because pubblished from 1947 to 2000

**- 338** duplicates

**- 2802** esophageal

**- 660** gastric

**- 826** barret

**- 194** case report

**1055** on head and neck and nutrition

**73 selected on abstract basis**

**- 982** excluded in abstract basis
